# North to south gradient and local waves of influenza in Chile

**DOI:** 10.1038/s41598-022-06318-0

**Published:** 2022-02-14

**Authors:** Christian Garcia-Calavaro, Lee H. Harrison, Darya Pokutnaya, Christina F. Mair, Maria M. Brooks, Wilbert van Panhuis

**Affiliations:** 1 Centro Programa de Salud Pública, Facultad de Ciencias Médicas, Universidad de Santiago, Avenida Libertador Bernardo O’Higgins no 3363, Estación Central, Santiago, Chile; 2 Center for Genomic Epidemiology, University of Pittsburgh, Pittsburgh, USA; 3 Department of Epidemiology, Graduate School of Public Health, University of Pittsburgh, Pittsburgh, PA USA; 4 Department of Behavioral and Community Health Sciences, Graduate School of Public Health, University of Pittsburgh, Pittsburgh, PA USA

**Keywords:** Infectious diseases, Epidemiology

## Abstract

Influenza seasonality is caused by complex interactions between environmental factors, viral mutations, population crowding, and human travel. To date, no studies have estimated the seasonality and latitudinal patterns of seasonal influenza in Chile. We obtained influenza-like illness (ILI) surveillance data from 29 Chilean public health networks to evaluate seasonality using wavelet analysis. We assessed the relationship between the start, peak, and latitude of the ILI epidemics using linear and piecewise regression. To estimate the presence of incoming and outgoing traveling waves (timing vs distance) between networks and to assess the association with population size, we used linear and logistic regression. We found a north to south gradient of influenza and traveling waves that were present in the central, densely populated region of Chile. Our findings suggest that larger populations in central Chile drive seasonal influenza epidemics.

## Introduction

Human influenza viruses persist as important disease-causing agents worldwide. Annually, influenza epidemics affect 5–10% of the world population and cause 250,000–500,000 influenza-associated deaths^1^. Influenza seasonality is influenced by complex interactions of biological, environmental, and social factors including viral mutations, temperature, humidity, rainfall, population crowding, and human travel^2–5^. Temperate regions experience peaks in influenza cases in the winter while tropical and subtropical areas are affected year-round with peaks occurring during the rainy season^1,6^. The virus travels from reservoirs from tropical to temperate regions and from high to low populated areas following human movement^5,7–13^. Identification of influenza seasonality is crucial for implementation of effective control strategies that can be tailored to local and national patterns^5,7,14^.

Previous influenza timing studies have used laboratory surveillance, mortality data, and symptomatic surveillance of influenza-like illness (ILI). While ILI surveillance has a high sensitivity and low specificity, it is a useful measure to describe influenza seasonality and identify influenza population patterns^15–21^. National surveillance is established globally; however, estimating influenza dynamics within countries requires substantial subnational and local datasets. High resolution data on the local level is essential to inform prevention measures such as vaccination campaigns and provider preparedness.

The majority of evidence supporting influenza timing comes from the developed countries and there are no studies of seasonal influenza timing in Chile. Chowell and colleagues found a south to north pattern of hospitalizations for H1N1 pandemic strain in 2009 in Chile that was later confirmed through mathematical simulations of the same data^22,23^. Influenza surveillance was enhanced after the 2009 pandemic with year-round surveillance of ILI in emergency departments (EDs), laboratory surveillance by sentinel providers, and surveillance of severe respiratory disease hospitalizations in public hospitals^24^. Here, we aimed to estimate the seasonality and spatial dependencies of influenza in Chile using ILI data reported in EDs.

## Results

### Hospitals included

**Figure 1:**
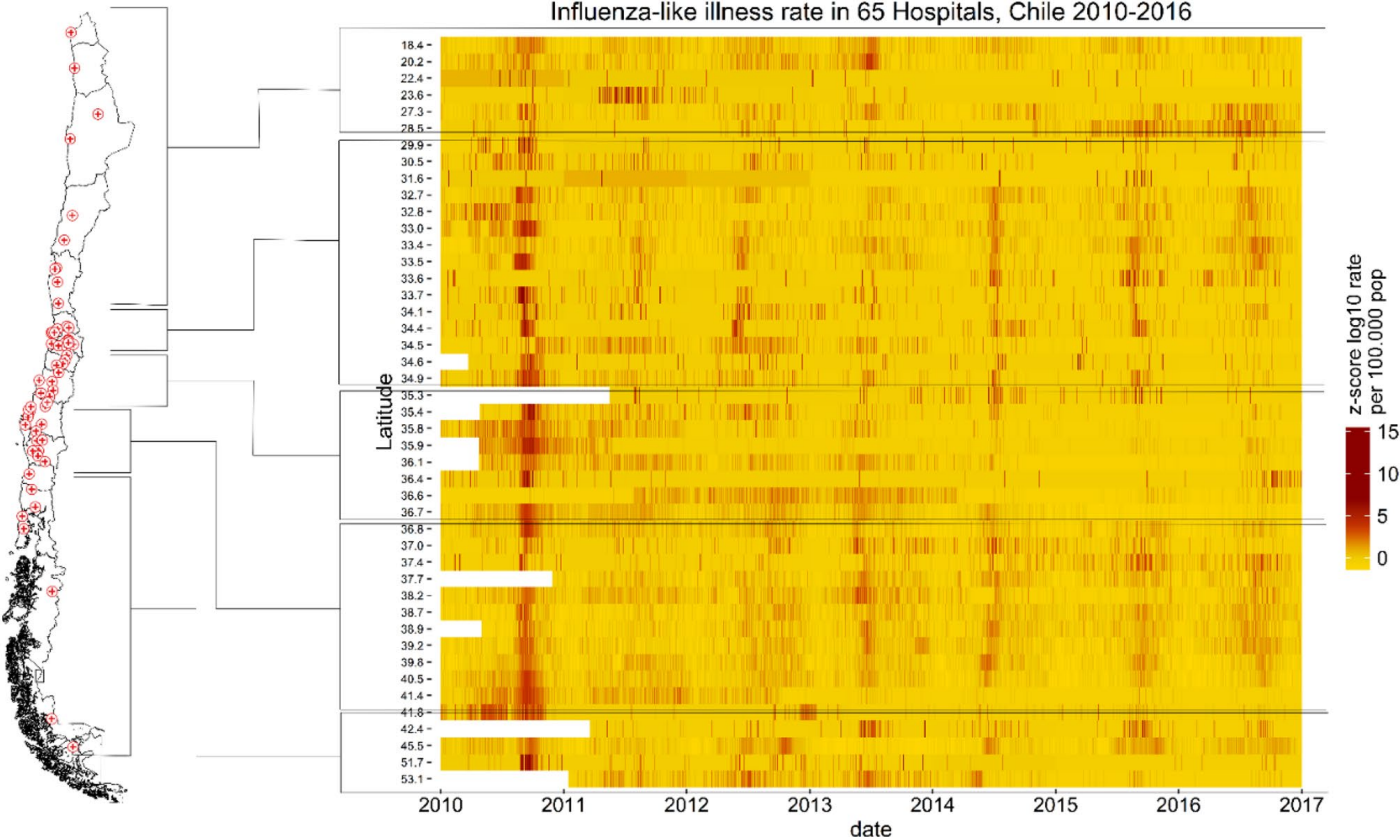
Hospital location and ILI rates, Chile 2010–2016*.* Left panel. Location of hospitals included through the Chilean territory. Right panel: Y axis, represent Hospitals ordered by latitude corresponding to the grouped zones marked by horizontal lines. X axis, time in days from 2010 to 2016. Gradient colors represent rates of ILI per 100,000 population. Z-score of log10 transformed shown for better visualization^24,27^.

From the 78 large and medium size hospitals in Chile, we excluded three hospitals from Santiago and one from Valparaiso with < 75% of daily ED data reported for the period between 2011 and 2016. Six pairs of hospitals from Santiago were combined, four corresponded to the merging of a pediatric and adult hospital (S2). We included 65 hospitals from the 15 regions and the 29 Chilean public health networks (Fig. 1).

A total of 242,982 ILI cases were reported from January 2010 to December 2016. Sixty-five percent of the days (n = 1.782.470) reported zero cases and 34% (n = 959,792) had ≥ 1 case reported. One percent (n = 1861) were days with missing data that ranged from 0 to 5% of the time series in different hospitals.

Influenza-like illness rates presented a cyclic pattern and synchrony across hospitals specially in 2010 with a spread pattern in the following years (Fig. 1).

### Predominant annual seasonality

**Figure 2:**
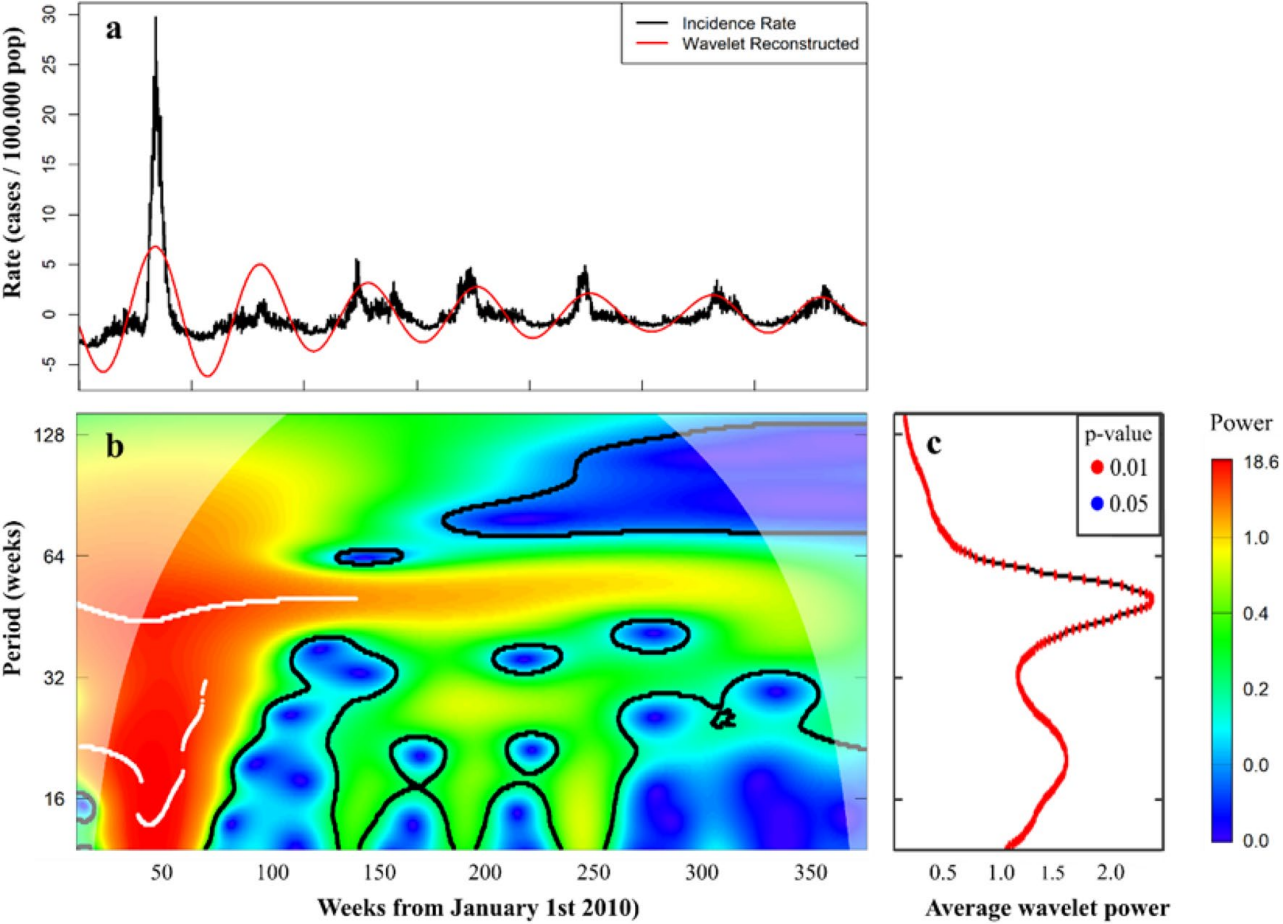
Detrended ILI incidence rate, wavelet reconstruction, local wavelet power spectrum and average power per period, Chile 2010–2016. (**a**) Detrended ILI incidence rate time series and wavelet reconstructed time series (red). Wavelet reconstruction using periods between 46 − 53 weeks, for Chile 2010–2016. (**b**) Local wavelet power spectrum of ILI rates for periods and time in weeks in Chile between 2010–2016. Significant power against white noise presented inside black contour. Ridge shown in white. A significant and high power was present through the complete time series for periods between 40 − 60. (**c**) Average power for periods, representing the average of local power from panel b with significance levels < 0.01 (red) and < 0.05 (blue)^24^.

We discovered a predominant annual seasonality for ILI rates in Chile. The annual pattern of ILI was present in the original time series and in the wavelet-reconstructed data (Fig. 2a). The local wavelet power spectrum showed an annual seasonality within periods from 40 to 64 weeks and the highest average power for period of 50 weeks (Fig. 2b and c, respectively).

**Figure 3:**
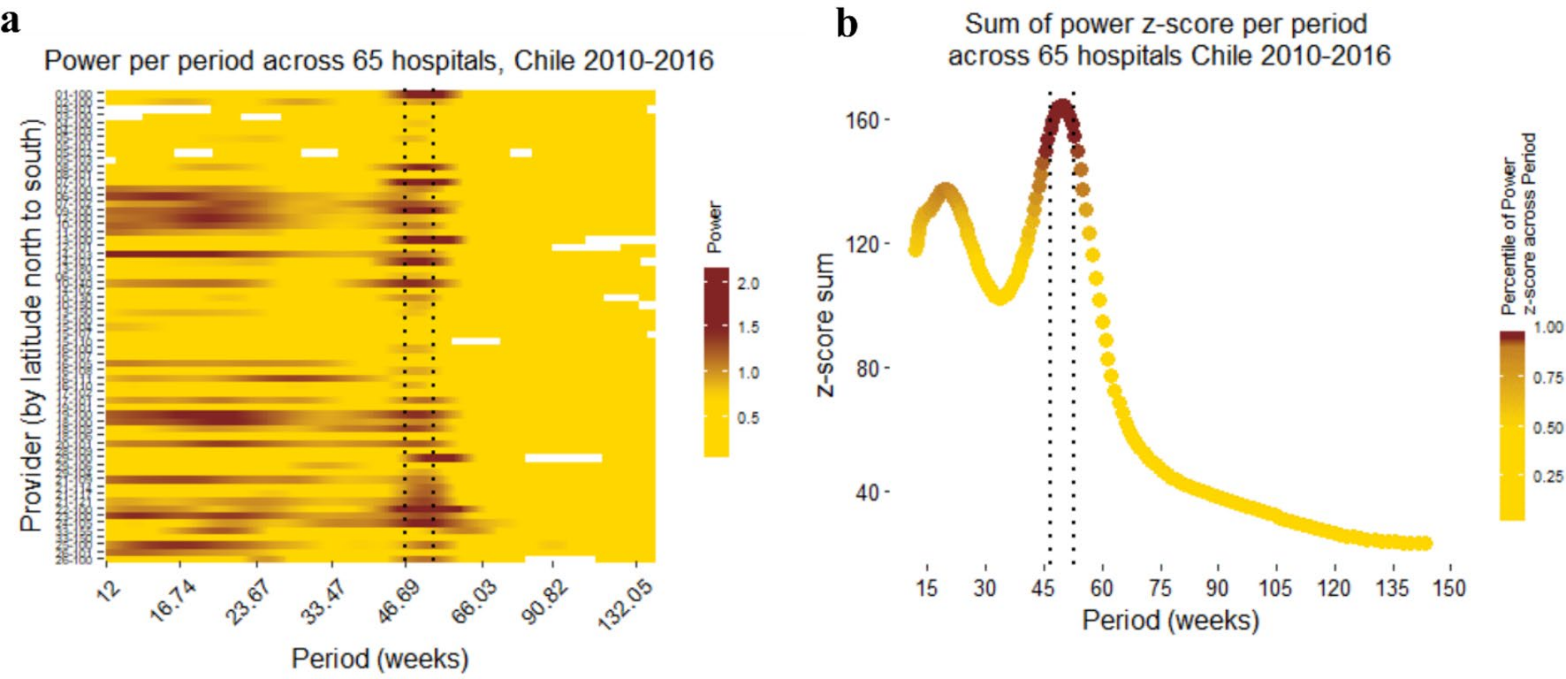
Power per period in hospitals and 95th percentile of power z-score across period and hospitals, Chile 2010–2016. (**a**) Power per each of the 65 hospitals presented across periods. Dark red represent higher power, yellow represents low power and fit, white spaces denote non-significant power compared to white noise. (**b**) Cumulative power z-score to calculate the periods with best fit across hospitals, represented as the 95th percentile of cumulative power. Power for each hospital were normalized, log transform, subtracted from the mean, and divided by the standard deviation. The sum of z-score for each period was plotted with the 95th percentile (dotted lines in both **a** and **b**)^24,27^.

The best seasonality, calculated as the 95th percentile of power, was observed between 46 and 53 weeks for all hospitals, health networks, and Chile overall (Figs. 2, 3, and S3 respectively)). Consequently, we used an annual periodicity from 46 to 53 weeks to filter the Morlet wavelet from each health network.

### Start and peak day of seasonal influenza in health networks

The median start and peak of the epidemic for all health networks and years was May 11th and August 4th, respectively (start day 131 of each year; IQR 117, 152 and peak day 217; IQR 200, 237, respectively). The median duration from start to peak was 84 days (IQR 82, 88), equivalent of 12 weeks or three months.

Despite the variability of start and peak day between and within health networks, there was consistency of timing of ILI across Chile. The duration of the epidemic was constant across health networks and latitudes (S4).

Influenza-like illness epidemics tended to start earlier than the previous year between 2010 and 2013, but from 2014 the start was delayed in each successive year. No significant association was found between the peak of the epidemic and predominant strain in the country for each year (S5).

### Population and a north to south pattern associated to early annual epidemics

**Figure 4:**
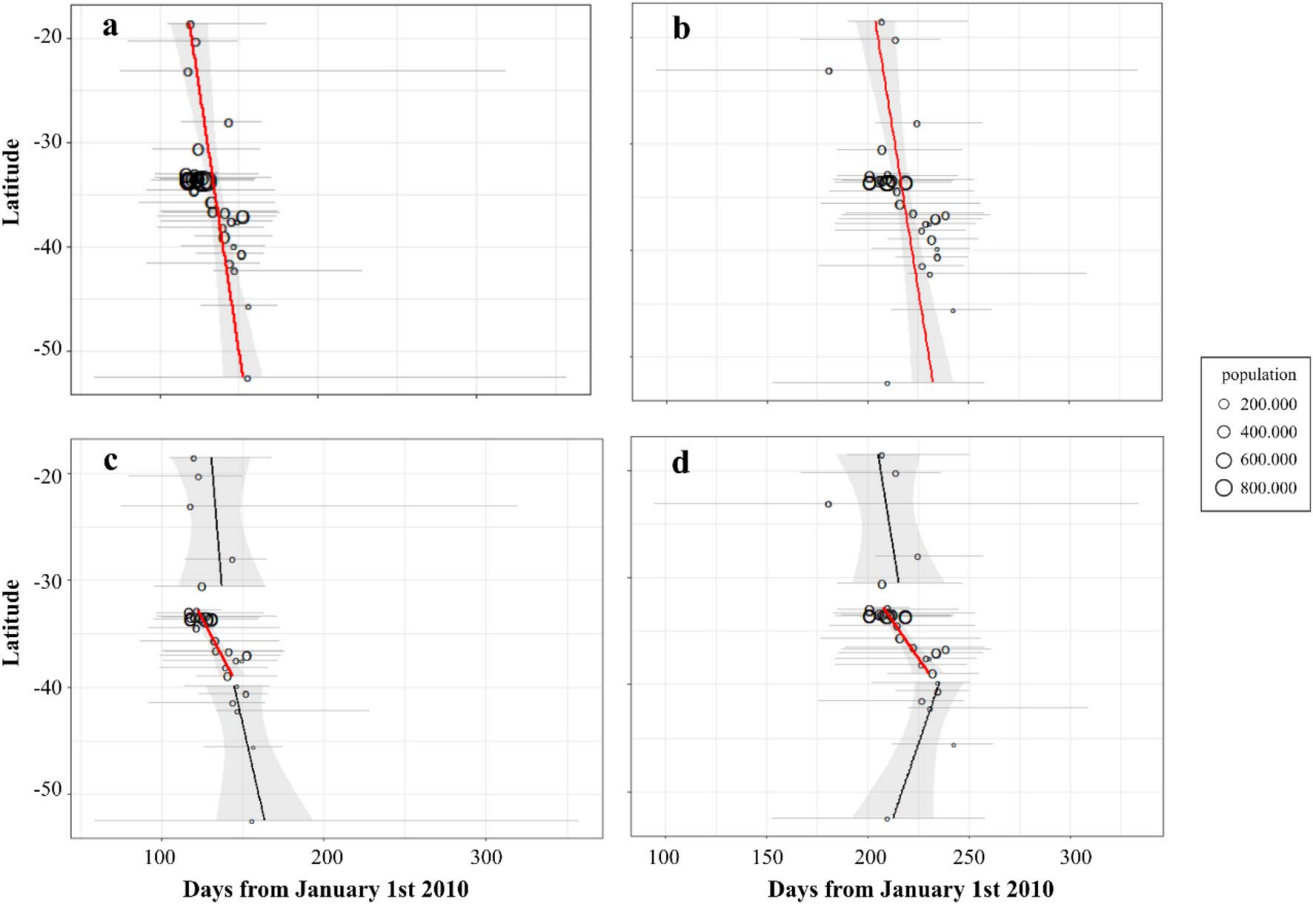
Start and peak of ILI vs latitude, 29 health networks in Chile, 2010–2016. Linear model for start day and peak day vs latitude. (**a)** and (b). all health networks; c and d health networks classified by zone: north, center, and south. Red lines: p-value < 0.05, black: p-value > 0.05. Vertical grey zones denote 95% CI, horizontal grey: range of start days for de period for each health network. Black circles size represents population size and black circle position, median start (panels **a** and **c**) or peak day (panels **b** and **d**). (**a**) Linear model for start day versus latitude denotes a slight, but significant north to south gradient: β = 0.99, *P* = 0.003, R^2^ = 0.04. (**b**) Linear model for peak day versus latitude denotes a slight, but significant north to south gradient: β = 0.8, *P* = 0.002, R^2^ = 0.04. (**c**) Linear model for start day versus latitude by zone shows a significant north to south gradient in the center zone of the country. North zone linear regression (top): β = 0.5, *P* = 0.75, R^2^ = 0.003. Center zone linear regression (middle): β = 3.49, *P* < 0.01, R^2^ = 0.09. Southern zone linear regression (bottom): β = 1.49, *P* = 0.33, R^2^ = 0.02. D) Linear model for peak day versus latitude by zone shows a significant north to south gradient in the center zone of the country. North zone linear regression (top): β = 0.8, *P* = 0.554, R^2^ = 0.01. Center zone linear regression (middle): β = 3.65, *P* < 0.01, R2 = 0.11. Southern zone linear regression (bottom): β = − 1.8, *P* = 0.08, R^2^ = 0.05^24,27^.

The univariate analysis showed that as latitude increased from north to south, the start and peak of the influenza season were delayed (Fig. 4). While there was a significant association between latitude and timing in the central zone of Chile (Fig. 4c and d), no association was present in the north or south of the country. There was a positive association between population size and earlier start and peak time of influenza. Given that influenza seasons vary annually, years was analyzed as a categorical variable and showed a significant association between start and peak day. Similarly, each year had a predominant strain in the country. The H3N2 strain had an earlier peak day and a shorter start-peak time compared to H1N1 strain. The presence of an airport in the territory of a health network was not associated with influenza timing (S6).

The best multivariate model for the outcome start day included nine variables: latitude, population, presence of airport, and years 2010 − 2016 encoded as dummy variables (R^2^ = 0.24; *P* < 0.01) (Table 1). A negative association was found between start date and population size (β = − 0.3; 95% CI, − 0.5 − −0.1; *P* < 0.01). Health networks with a nearby airport was associated with an estimated delay of 9.5 days for the start of an ILI epidemic (95% CI, 0.84 − 18.19; *P* < 0.05). A north to south pattern was found across the country, for one degree increase in latitude there was one day delay on the start of ILI (β = 0.903; 95% CI 0.315 − 1.491; *P* < 0.01) (Eq. 1 and Table 2).

1 $$ Sd = 170.9 + 0.9\left( {Lat} \right) - 0.31\left( {Pop10,000} \right) + 9.5\left( {Airport} \right) + 49.7 \left( {Y2010} \right) + 30.5\left( {Y2011} \right) + 12.9 \left( {Y2012} \right) + 14.8\left( {Y2014} \right) + 23.5\left( {Y2015} \right) + 23.3(Y2016) $$

where *S*_*d*_ = Start day from January 1st from each year; *Lat* = South Latitude*; Airport* = Presence of an airport*; Y* = Dummy for the year noted.

**Table 1:** Multivariate regression model selection for influenza-like illness start day and peak day, using best subset method.

Model^‡^	# of Variables	Variables used*	Start day models				Peak day models			
			R^2^	Adjusted R^2^	Cp	BIC	R^2^	Adjusted R^2^	Cp	BIC
A	7	year, latitude	0.24	0.21	16.0	− 13.0	0.41	0.39	11.0	− 65.0
B†	8	year, latitude, population	0.26	0.23	12.0	− 14.0	0.43	0.40	8.4	− 65.0
C^††^	9	year, latitude, population, airport	0.28	0.25	9.0	− 14.0	0.43	0.40	9.0	− 61.0

^‡^ Best model for all possible models with the given numbers of variables using RSS.

*Year was considered as 6 dummy variables and was forced in. Strain was excluded given the high correlation with year.

^†^ Selected peak day model.

^††^ Selected start day model.

**Table 2:** Selected multivariate models for ILI start day and peak day.

	Start day model				Peak day models			
	β	95% CI	*p *value	Adjusted R^2^	β	95% CI	*p *value	Adjusted R^2^
Model			< 0.01	0.2471			< 0.01	0.4016
**Variables**								
Latitude	0.903	0.314 , 1.490	< 0.01		0.780	0.345 , 1.214	< 0.01	
Population (10,000)	− 0.313	− 0.519 , − 0.105	< 0.01		− 0.166	− 0.316 , − 0.016	0.03	
Airport (yes)	9.519	0.847 , 18.190	0.03		–	–		
**Year**			< 0.01				< 0.01	
2010	49.710	34.892 , 64.518			47.810	36.861 , 58.756		
2011	30.500	15.687 , 45.313			28.710	17.758 , 39.653		
2012	12.960	− 1.850 , 27.773			11.800	0.8581 , 22.751		
2013 (Ref.)	–	–			–	–		
2014	14.860	0.0508 , 29.674			− 1.085	− 12.03 , 9.8615		
2015	23.520	8.7108 , 38.334			31.590	20.642 , 42.535		
2016	23.310	8.4998 , 38.123			20.940	9.9897 , 31.882		

The best model for ILI peak included eight variables: latitude, population and year as a dummy variable (R2 = 0.40; P < 0.01) (Table 1). A north to south pattern was found for latitude (β = 0.780; 95% CI, 0.346 − 1.215; *P* < 0.01) and a negative association between ILI peak and population size (β = − 0.16, 95% CI − 0.366 to − 0.016; *P* < 0.05) (Eq. 2 and Table 2).

2 $$ Pd = 173.43 + 0.78\left( {Lat} \right) - 0.16\left( {Pop10,000} \right) + 47.8 \left( {Y2010} \right) + 28.7 \left( {Y2011} \right) + 11.8 \left( {Y2012} \right) - 1.09 \left( {Y2014} \right) + 31.59 \left( {Y2015} \right) + 20.94 (Y2016) $$

where P_*d*_ = Peak day from January 1st from each year; *Lat* = South Latitude*; Y* = Dummy for the year noted.

### Local travelling waves

**Figure 5:**
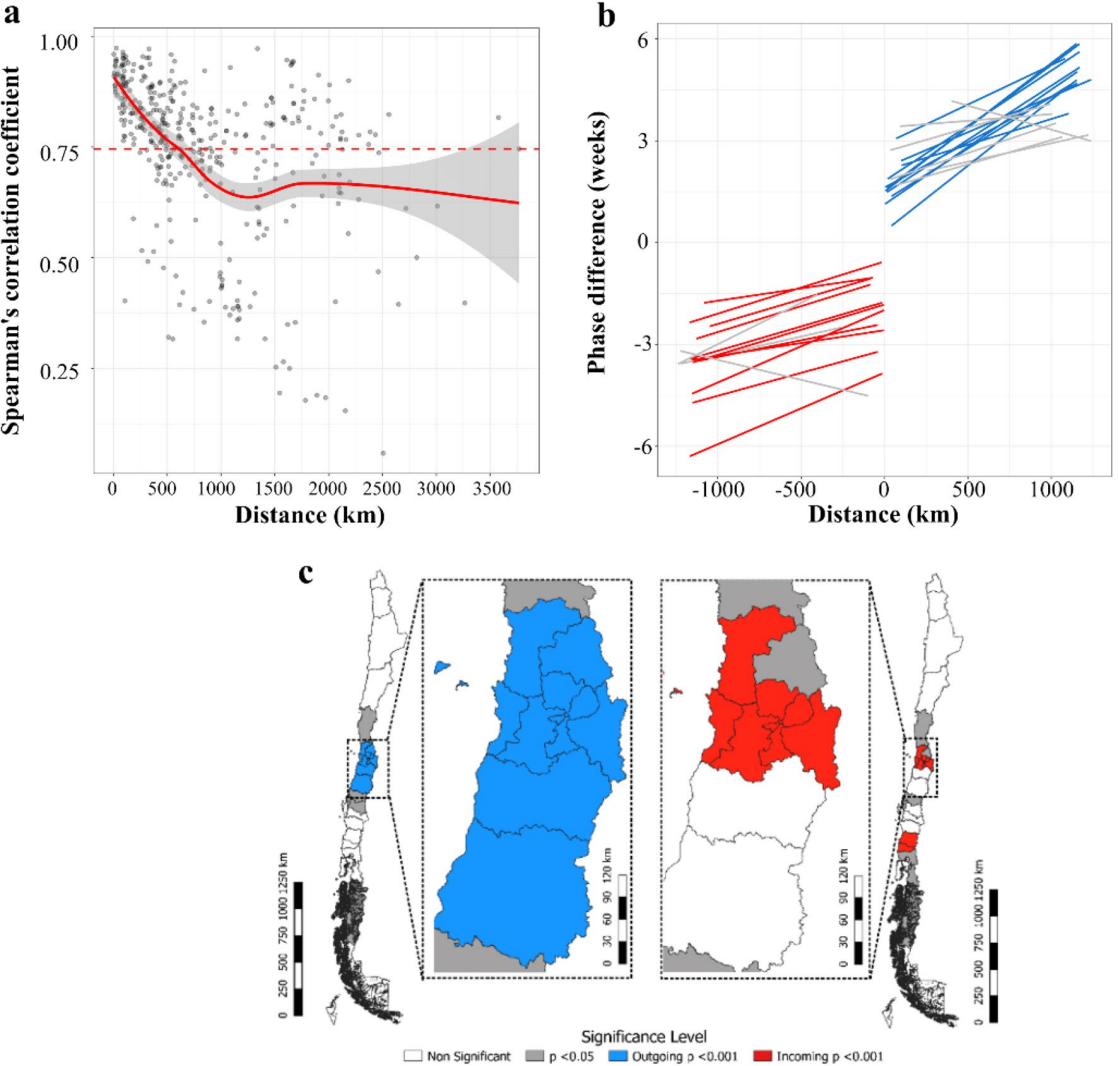
Distance vs pairwise Spearman's correlation and travelling waves of influenza in health networks. Chile 2010–2016. (**a**) Spearman’s correlation of phase angles versus distance between all pairs of health networks. Dashed lines represent the average correlation. The model used a loess model with α = 0.75 (red line) and 95% CI (grey). There is a change in the descending relation of correlation and distance at 1250 km that we used as the upper distance limit to define local wave. (**b**) Phase difference in weeks vs distance (km) for Health Networks with incoming (red) and outgoing (blue) waves. In grey linear models with a significance level of 0.05. In red and blue, linear models with a Bonferroni corrected significance level < 0.001. The distance sign was inverted for negative lag times for a more intuitive display of incoming waves. (**c**) Geographic location of health networks with significant traveling waves. Incoming and outgoing waves are clustered in the center of the country. Colors correspond to panel (**b**)^24,27^.

We used a second-degree local polynomial regression model (LOESS) to determine the distance limit for the local traveling wave models. We found a descending Spearman’s correlation of phase angles up to 1250 km that disappeared for larger distances (Fig. 5a) in both the reconstructed wavelet analysis and in the original rate time series data (S7). As a result, we included health networks closer than 1250 km in each model of local traveling waves.

There were 11 health networks with outgoing traveling waves and 10 that presented incoming waves located at the center and southern regions of Chile (*P* < 0.0017) (Fig. 5b and c). Local traveling waves were associated with population size after adjusting for latitude. For every 10,000 individuals, a health network had a 23% greater odds of presenting local outgoing waves (OR = 1.23; 95% CI 95, 1.05 − 1.44; *P* = 0.001) and 18% higher odds of presenting incoming waves (OR = 1.18; 95% CI, 1.03 − 1.34; *P* = 0.01) (S8). Both logistic regression models had excellent discrimination for outgoing and incoming waves (AUC = 0.914, 95% CI 0.8 − 1.0 and AUC = 0.884, 95% CI 0.729 − 1.0, respectively).

## Discussion

We found a predominant annual seasonality of influenza in Chile with a periodicity of 50 weeks (95% CI, 43 − 53). This period coincided with the annual seasonality of influenza reported from other temperate territories and from the Chilean Ministry of Health influenza surveillance reports^48^. To our knowledge, this is the first study that confirms a cycle of seasonal influenza in Chile.

There was a north–south latitudinal gradient for the start and peak of influenza related to the central zone between latitudes 31 and 40 degrees south. Chowell and colleagues found a south to north hospitalization gradient for the H1N1 2009 pandemic^22^. The difference between Chowell results and our findings can be explained by the annual variability of influenza epidemics due to weather, population immunity, or human movement^49^. We did not include 2009 data in our analysis; however, we found a south-north latitudinal gradient in 2015 for the start date of the ILI season (S9). In the 2009 and 2015 epidemics, the first influenza cases were reported in southern cities with populations greater than 200,000. While health networks consisting of large populations from central Chile have a higher probability of reporting the first cases of influenza, southern zones with relatively large populations can also experience epidemics.

Population size was also associated with the start and peak of influenza epidemics as described previously in Perú, Hong Kong, Brazil and the USA^5,7,12,13,21^. The patterns found were mostly influenced by the center region of Chile which includes Santiago, the nation’s capital and most populated city. The outward, rapid spread of influenza from Santiago to the rest of the country has been previously reported by Burger et al. in Chile and are similar to the findings described by Viboud et al. in the USA^5,23^. These findings are supported by the association between population and the presence of local incoming and outgoing wave. Health networks with significant local outgoing and incoming traveling waves were primarily located in central Chile; two networks with significant incoming waves were also located in the southern region. Our findings suggest that larger populations located in the center of the country drive seasonal influenza epidemics. Health networks in the central zone spread influenza to neighboring networks through outgoing traveling waves. Networks receive incoming waves in the same zone and in southern Chile.

This study has several limitations. We assumed that all hospitals had the same age distribution and did not adjust for age structure of health networks. Controlling for age may provide more detailed information on influenza transmission in future studies. We did not include ED data from private insurance companies, hospitals, or practices because they were not available; however, public insurance covers 74% of the population in Chile^26^. Additionally, we included data from hospitals located in a range of latitudes that are collected through a centralized reporting system with national guidelines^24^. While we used secondary ILI case data from ED reports that were based on clinical diagnosis without laboratory confirmation, laboratory surveillance is available only for 25 hospitals with partial coverage of the countries´ territory. ILI has been reported as a useful measure to describe influenza seasonality and identify influenza population patterns^21^. On the other hand, ILI surveillance operates in all Chilean hospital EDs year-round resulting in more complete seasonality data. We assumed a constant proportion of emergency consultation to other providers. Private hospitals, private practices, and public primary healthcare centers could attract patients with public insurance, especially in seasons of high demand. We assessed this limitation by estimating the population with public insurance that lives in a 5 km radius that are more likely to visit a hospital ED compared to a public or private provider. Instead of assuming an annual seasonality, we estimated the best seasonality before fitting our models. We selected the best models for the start and peak of influenza by comparing all possible models.

This is the first study to estimate seasonal influenza seasonality in Chile and its patterns across latitudes. Novel strains should be considered when different patterns are detected. If a novel strain is detected early, patterns could differ from the ones found here.

Our findings can help decision-makers to prepare for the influenza season by prioritizing zones for early vaccination campaigns, reallocating hospital beds for annual epidemics, and distributing resources according to patterns of influenza. Zones with larger populations and those located in the center of the country would require earlier implementation of interventions to reduce the spread of influenza to regions across the country.

## Methods

### Data sources

We obtained daily ILI case data collected by the Chilean Ministry of Health between 2010 and 2016 from public hospitals EDs. ILI is an acceptable measure that is correlated to laboratory surveillance regarding population patterns^21^. We used ILI from ED rather than sentinel laboratory surveillance data given that influenza surveillance is limited to 25 hospitals in the country with limited territorial coverage. We supplemented gridded population data from WorldPop with official governmental data on hospital locations, census information, and population coverage to estimate the population with access to each hospital^25–28^.

We excluded community hospitals, hospitals without an ED, and hospitals that reported < 75% of daily ED data between 2011 and 2016 (< 1920 days). Hospitals that had ≥ 5% missing data in groups of 20 consecutive days were also excluded to reduce imputation errors. We assumed missing data were missing at random. Twelve different imputation methods were tested using randomly generated missing data compared to the original data. Time series imputation with Kalman Smoothing showed the best results for estimating seasonality compared to the original data^29,30^.

### Data preparation

Given that patients experience a decline in ED access with increasing distance, we chose a five kilometer radius to estimate the population covered by each hospital^31,32^. We obtained the population estimates per 100 × 100 m grid from WorldPop for 2010 and 2015 and adjusted by census data for each municipality and year^28^. We used linear interpolation to impute population estimates for 2011 − 2014, and 2016. For each hospital coverage area, we adjusted for the proportion of individuals with public insurance for each municipality per year and calculated daily rates of ILI over the estimated population^26^. Data from hospitals in the same health network and within 5 km from each other were combined into one time series. To determine seasonality across health networks, we calculated the ILI rate by network by adding ILI and population data from all hospitals within each health network. We used a mid-point location from hospitals within each network to determine the health network’s latitude.

### Data transformation using wavelets

We used wavelet analysis to determine the best periodicity (e.g. seasonality) of influenza across 29 Chilean health networks. Wavelet transformation is an adequate method to determine seasonality for non-stationary time series and to determine seasonality of a periodic time series without assuming a priori a specific seasonality^33^. Wavelet transformation corresponds to the cross-correlation of ILI rate’s time series with wavelets of different “widths” or “scales” across time^33^. Morlet wavelet has been used to calculate infectious disease rates for dengue, pertussis, measles, and influenza^34–39^. We computed the Morlet wavelet transformation for the entire country, each health network, and each individual hospital^34,35,40^.

We conducted wavelet transformations for periods (scales) from 14 − 144 weeks. Statistical significance was tested by comparing the wave signal against 1000 randomly generated time series (i.e. white noise) with a significance level of 0.05^41^. The local wavelet power spectrum was used to visualize the cross-correlation between rates and each group of wavelets (Fig. 2b, S1 and Equation s1)^33,42^.

To determine the best seasonality (periods with higher power), we computed the average power for each period, over all time points, from the local wavelet power spectrum for Chile, each health network, and for individual hospitals, according to the work of Torrence^42^:

3 $$ \overline{W}^{2} \left( s \right) = \frac{1}{N} \mathop \sum \limits_{n = 0}^{N} \left| {Wn\left( s \right)} \right|^{2} $$

where *N* represents the number of observations.

We defined the best seasonality of influenza as the range of periods within the 95th percentile of average power across periods.

### Association between epidemic timing and latitude in health networks

To determine the association between influenza timing and latitude, we selected the wavelet transform using the range corresponding to the best seasonality for each health network. We reconstructed the epidemic cycles using the filter defined by Torrence and Campo (S1 and Equation s2)^42^. For each health network, the start date of an annual ILI epidemic was defined as the date when the reconstructed cycles reached the midpoint between the lowest point and the peak of the signal for each year from 2010 to 2016. We calculated the peak day for the corresponding period along with the median start and peak date for each health network.

We developed univariate, piecewise, and multivariate linear regression models to assess the relationship between the predictor, latitude, and start and peak outcomes of the epidemic across the health networks. For the piecewise models, we separated the country into three zones: north, center, and south with south latitude ranges of < 31, 31–40, and > 41, respectively. For multivariate analysis, we included latitude and population as continuous variables, a binary variable to describe the presence of an airport within the health network territory, and dummy variables for years 2010 to 2016 with 2013 as the reference. The predominant influenza strain was excluded due to linear dependencies with years 2011 and 2014. The final multivariate model was selected by testing all possible predictor combinations with the regression sum of squares method and by comparing the BIC and Mallow’s Cp^43^.

### Local traveling waves of influenza

A Morlet wavelet is a complex function with a real and an imaginary part. The real part enables separation of the amplitude and extraction of the phase of a signal as the timing within a specific period, regardless of the amplitude^33,42,44^. Phases are continuous cycles that are presented as phase angles in radians from -π to + π^41^. We extracted phase angles using a band of periods within the 95th percentile of power as described previously by Torrence (S1 and Eq. 3).

In order to define a local traveling wave, we used a second-degree local polynomial regression model (LOESS) with Spearman’s correlation between pairs of health networks phase angle time series to distance with parameter α = 0.75. Then, we determined the distance were the smallest correlation after an initial linear segment of the LOESS model was found. All pairs of health networks within this distance limit were included for each of the 29 linear models for traveling waves.

We calculated the phase angle (timing) time-series median difference for all possible pairs of health networks and tested the association to distance. The sign of the phase difference represented the relative timing of ILI. A negative difference indicated a delayed timing of influenza (incoming); positive difference indicated outgoing travelling waves. We fitted two linear models for each health network to all others, one for positive phase differences and the second for negative phase difference:

4 $$ \theta _{{p,q}}  = \left\{ {\begin{array}{*{20}c}    {C - \beta _{p} d_{{p,q}} \quad for \quad \theta  < 0,\quad incoming\;waves}  \\    {C + \beta _{p} d_{{p,q}} \quad for \quad \theta  > 0, \quad outgoing\;waves}  \\   \end{array} } \right\} $$

where *θ*_*p,q*_ is the lag between health networks *p* and *q* and *d*_*p,q*_ is the distance in kilometers between health networks. The distance sign was inverted for negative lag times for a more intuitive display of incoming waves. We considered a local traveling wave as a significant linear association between phase difference and distance using a corrected Bonferroni significance level of 0.0017.

We developed separate logistic regression models for incoming and outgoing waves to determine the association between traveling waves and population estimates, adjusting for each health network latitude. To evaluate model discrimination, we used the receiver operating characteristic curve and the area under the curve (AUC)^45^.

We used R version 3.6.2 R package Leaps for model selection, and R package WaveletComp for wavelet transformation and phases extraction^46,47^.

### Previous presentations

This work has not been previously presented or published.

## Supplementary Information

Supplementary Information.
