# Supplementary material for: North to south gradient and local waves of influenza in Chile
Source: Sci Rep. 2022 Feb 14;12:2409. doi: 10.1038/s41598-022-06318-0 (PMC8844068; doi:10.1038/s41598-022-06318-0)
Supplement: Supplementary file 1 — Supplementary Information. [file 41598_2022_6318_MOESM1_ESM.docx]

**Supplementary Material**

**S1. Methods**

We conducted an ecological study to assess the seasonality of influenza in Chile and the relationship with local influenza patterns. We first collected time-series data from daily ED visits and calculated health network-specific rates of ILI. Second, to test the hypothesis that there is an annual seasonality of ILI, we estimated the seasonality of ILI across health networks using wavelet analysis. Then, to test the hypothesis that annual epidemic start and peak are associated to latitude, we extracted the annual start and peak dates within each health network and assessed the relationship between the start and peak of ILI and latitude using a linear and piecewise regression. Finally, to test the hypothesis that there are local travelling waves associated to population, we estimated the relation between timing versus distance represented as incoming and outgoing travelling waves for each health network using linear regression and the relation to population using a logistic regression model.

**Data transformation**

For all transformations, we used the same parameters. We used a non-dimensional frequency ω_0_ = 6, that has shown a good performance and good resolution and has been used previously for infectious disease [1, 2]. We used periodicity step size δj of 7 on a linear scale that represents a weekly resolution using daily data. For each scale s and time interval δt, the continuous wavelet transform of a time series *x_k_* is defined as [1]:

Equation s1

$$Wn\left( s \right)= \sum_{k=0}^{N-1} xk\psi* \frac{(k-n)\delta t}{s}$$

Where ψ* represents the complex conjugate Morlet wavelet. N represents the time index, ranging from zero to the total number of time points, and s are the scales.

We reconstructed the epidemic cycles using a filter defined by Torrence and Campo [1]

Equation s2

$$x_{n}^{'}=\frac{\delta t\sqrt{\delta t}}{C_{\delta}\psi_{0}} \sum_{j=j1}^{j2} \frac{\mathfrak{R\{}W_{n}(sj)\}}{\sqrt{sj}}$$

Where δt, the periodicity step size was 7 and C_δ_, the reconstruction factor, was 0.776 empirically derived for the non-dimensional frequency ω_0_ = 6 [1]. Values from j1 and j2 were the lower and upper limits of the range of periods that included the 95^th^ percentile of power.

A Morlet wavelet is a complex function with a real and an imaginary part. The real part enables to separate the amplitude and extract the phase of a signal as the timing within a specific period, regardless of the amplitude [3, Torrence, 1998 #42, Grinsted, 2004 #67]. Phases are continuous and are presented as phase angles in radians from -π, the starting point, to +π before resetting and starting a new cycle [4]. We extracted phase angles for each Health Network as described previously by Torrence [1, 5, 6]:

Equation s3

$$\phi_{n}(s) = {tan}^{-1} \left( \frac{\mathfrak{I \{}W_{n}^{XY} (s)\}}{\mathfrak{R \{}W_{n}^{XY} (s)\}} \right)$$

Where ϕn(s) is the phase for a scale s, ℑ{W_n_^XY^(s)) is the imaginary part, and ℑ{W_n_^XY^(s)), the real part.

**Results**

**S2. Selected and combined hospitals in Santiago, Chile 2011-2016**


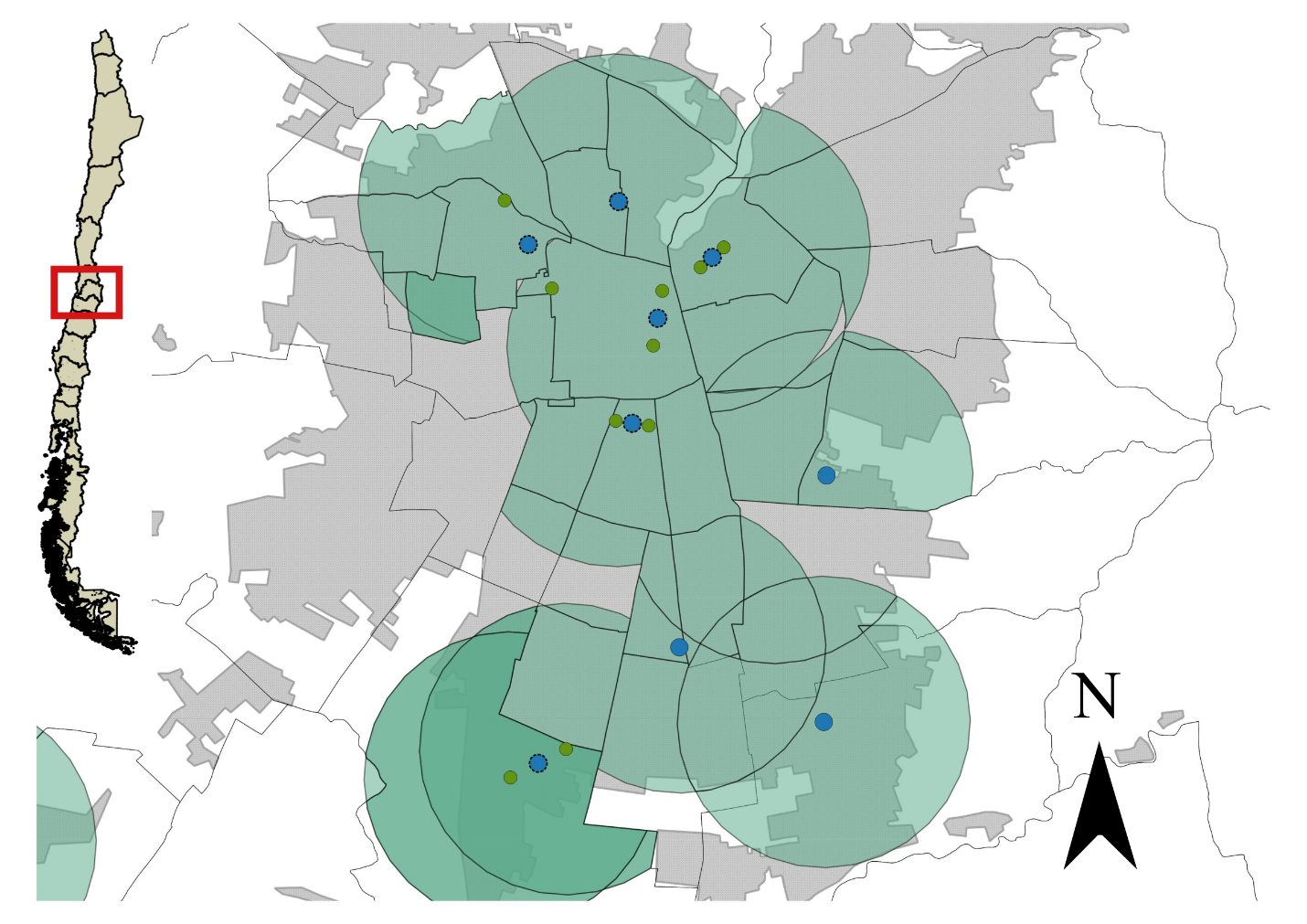
Selected hospitals from Santiago.

Blue dots: Hospitals included. Green dots: hospitals that were combined with the nearest green point to form the blue dot between both green hospitals. † Pediatric hospital that were combined, * Adult Hospital that were combined. The upper blue dot with † and * represent the combination of two neighboring hospitals.

Map created using the Free and Open Source QGIS 3.0 Girona available from <https://qgis.org/en/site/index.html>.

Data available from the National Catalog of Geospatial information, Ministry of National Assets available from: <http://www.geoportal.cl/geoportal/catalog/main/home.page>

**S3. Local Wavelet Power Spectrum of ILI rates for the 29 Health Networks, Chile 2010-2016 (Codes include 1 to 33 excluding codes 27, 30,31,32). Significant power against white noise inside black contour. Ridge in white. A significant and high power was present in most of the Health Networks through the complete time series for periods between 40 and 60 weeks.**


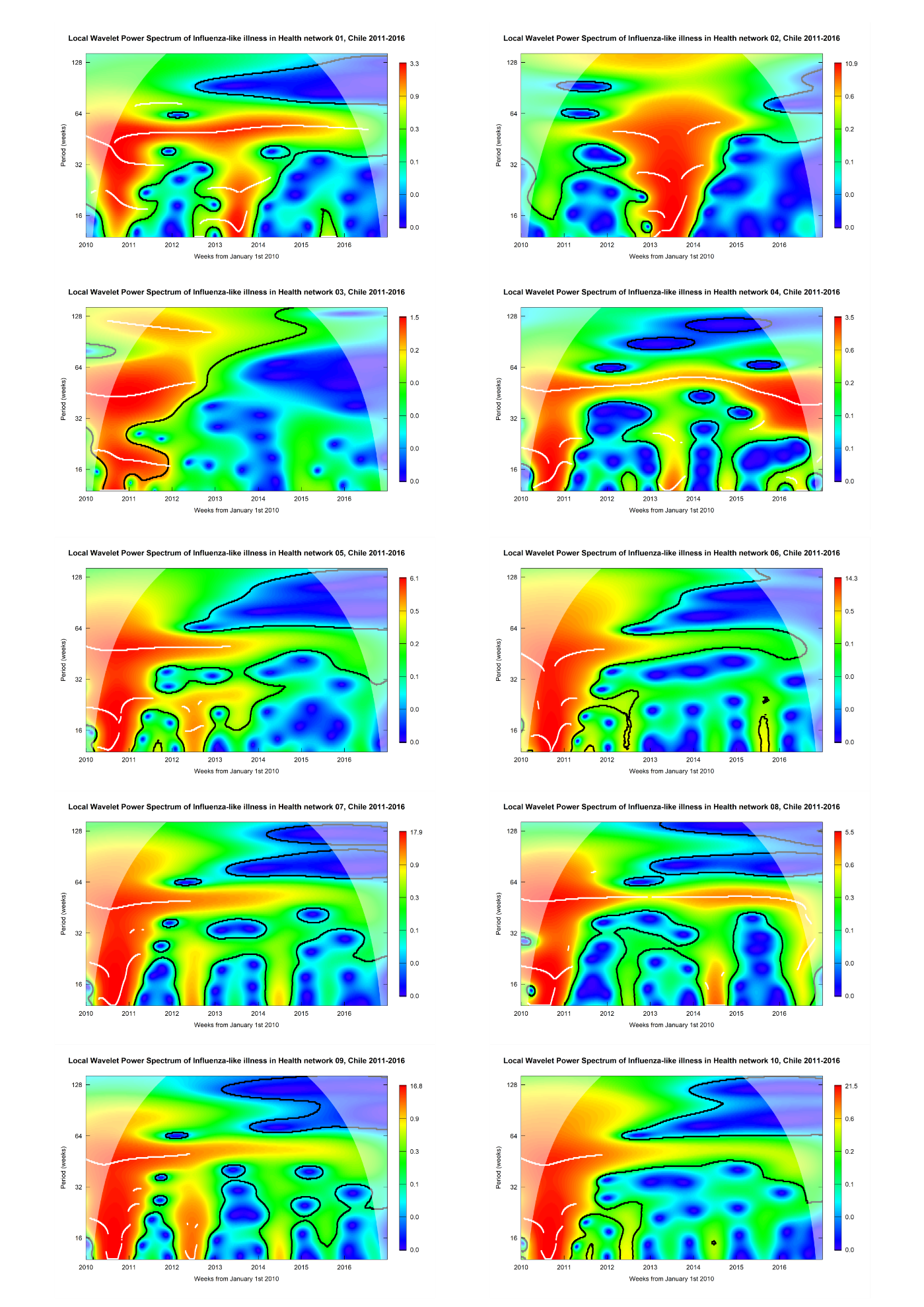


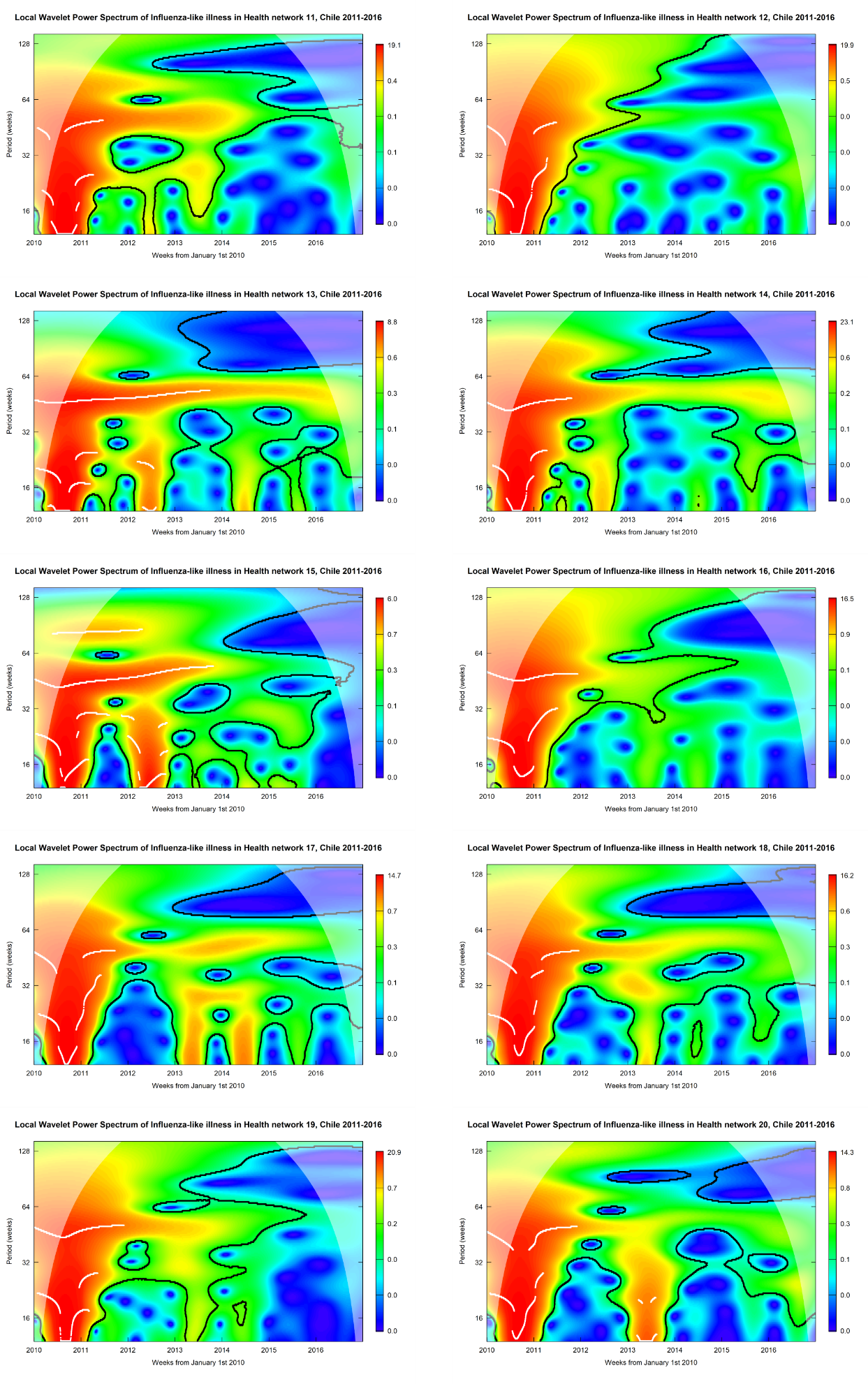

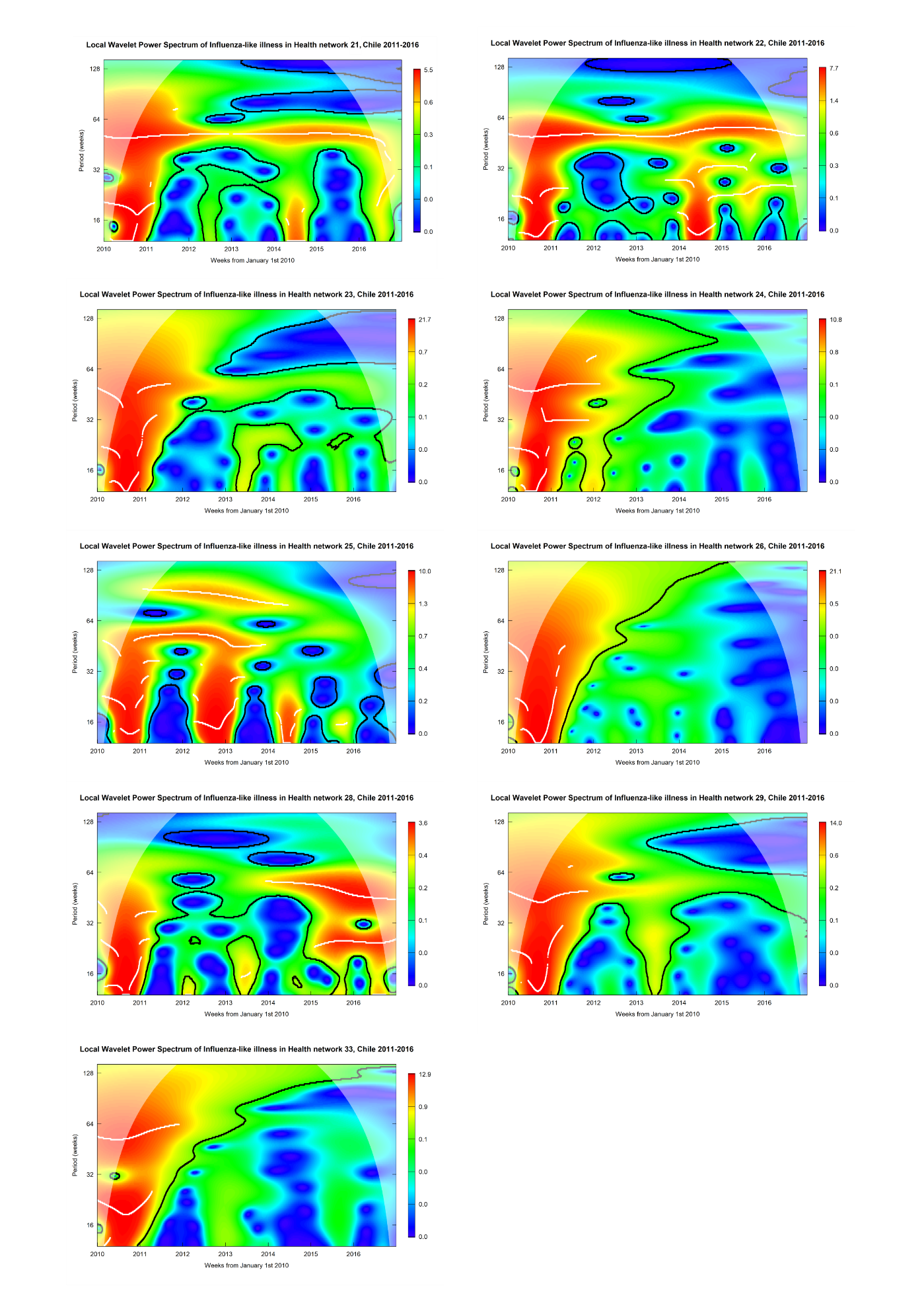


**S4. Start and peak of ILI in emergency room by Health Service and Latitude, Chile 2010-2016**


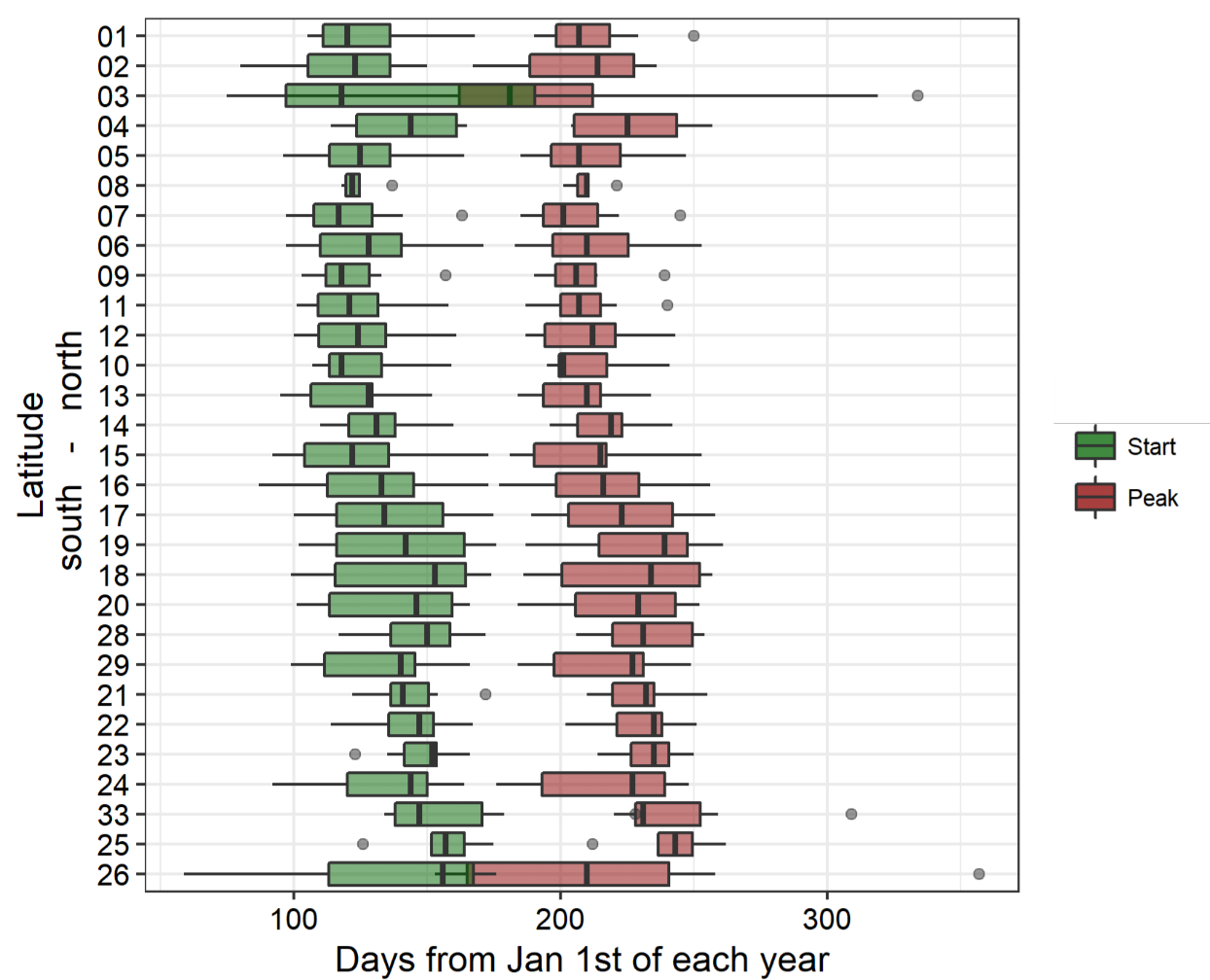


**S5. Start of influenza season and predominant strain per year in 65 hospitals, Chile 2010-2016**


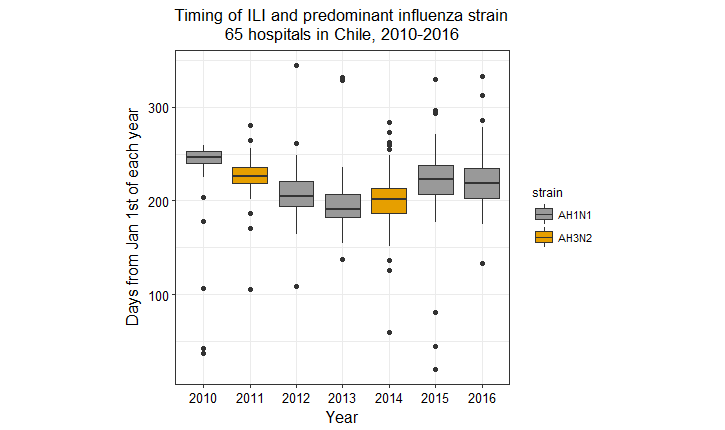


**S6. Univariate regressions for start day, peak day**

|  | Start day | | | |  | Peak day | | | |
| --- | --- | --- | --- | --- | --- | --- | --- | --- | --- |
|  | β | Std.Error | p-value | Adjusted R2 |  | β | Std.Error | p-value | Adjusted R2 |
| Latitude | 0.9962 | 0.334 | 0.003 | 0.03761 |  | 0.8443 | 0.2766 | 0.003 | 0.03954 |
| Year (continuous) | -3.331 | 1.135 | 0.004 | 0.0363 |  | -3.1724 | 0.9347 | 0.001 | 0.0495 |
| Population | 0.000 | 1.158E-05 | 0.006 | 0.032 |  | 0.000 | 9.66E-06 | 0.027 | 0.019 |
|  |  |  |  |  |  |  |  |  |  |
| Year |  |  |  | 0.171 |  |  |  |  | 0.3475 |
| 2010 | 50.000 | 7.88 | 0.000 |  |  | 47.966 | 5.796 | 0.000 |  |
| 2011 | 30.793 | 7.88 | 0.00013 |  |  | 28.862 | 5.796 | 0.000001 |  |
| 2012 | 13.069 | 7.88 | 0.09883 |  |  | 11.862 | 5.796 | 0.042022 |  |
| 2013 | - | - |  |  |  | - | - | - |  |
| 2014 | 14.828 | 7.88 | 0.061376 |  |  | -1.103 | 5.796 | 0.849200 |  |
| 2015 | 23.517 | 7.88 | 0.003 |  |  | 31.586 | 5.796 | 0.000000 |  |
| 2016 | 23.172 | 7.88 | 0.004 |  |  | 20.862 | 5.796 | 0.000404 |  |
|  |  |  |  |  |  |  |  |  |  |
| Strain |  |  |  | -0.005 |  |  |  |  | 0.01533 |
| AH1N1 | - | - | - |  |  | - | - | - |  |
| AH3N2 | 0.8586 | 5.1327 | 0.867 |  |  | -8.576 | 4.212 | 0.0431 |  |
|  |  |  |  |  |  |  |  |  |  |
| Airport |  |  |  | 0.00311 |  |  |  |  | 0.003991 |
| No | - | - | - |  |  | - | - | - |  |
| Yes | 6.374 | 4.992 | 0.203 |  |  | 1.844 | 4.153 | 0.658 |  |

**S7. Distance vs pairwise Spearman's correlation of ILI rate, phase angles and wavelet reconstruction. Health Networks, Chile 2010-2016 (n = 406 pairs)**


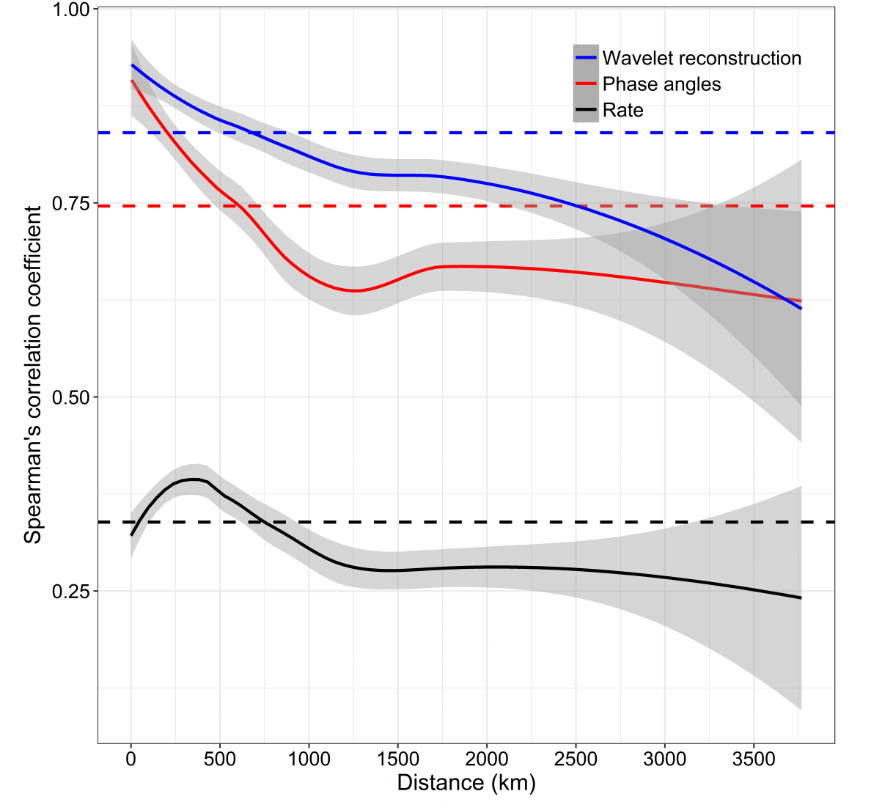


Spearman’s correlation versus distance between pairs of Health Networks. Wavelet reconstructed time series (blue), phase angle time series (red), rates (black). Dashed lines represent the average correlation. The 3 models used a loess model with α = 0.75 and 95% CI (grey). Different correlations and patterns showed a change at the distance of 1250 km that we used as the upper distance limit to define local wave.

**S8. Logistic models for outgoing and incoming waves in Health Networks, Chile 2010-2016**

|  | Outgoing Waves | |  | Incoming Waves | |
| --- | --- | --- | --- | --- | --- |
|  | OR | CI 95% |  | OR | CI 95% |
| Variables |  |  |  |  |  |
| Population (10,000 ) | 1.23* | 1.05,1.44 |  | 1.18* | 1.03,1.34 |
| Latitude (south) | 0.95 | 0.78,1.15 |  | 1.08 | 0.86,1.36 |
|  |  |  |  |  |  |
| AUC | 0.914 | 0.800, 1.00 |  | 0.884 | 0.729, 1.00 |
| * p < 0.05 | | | | | |

**S9. Start of ILI vs Latitude per year in 29 Health Networks, Chile 2010-2016**


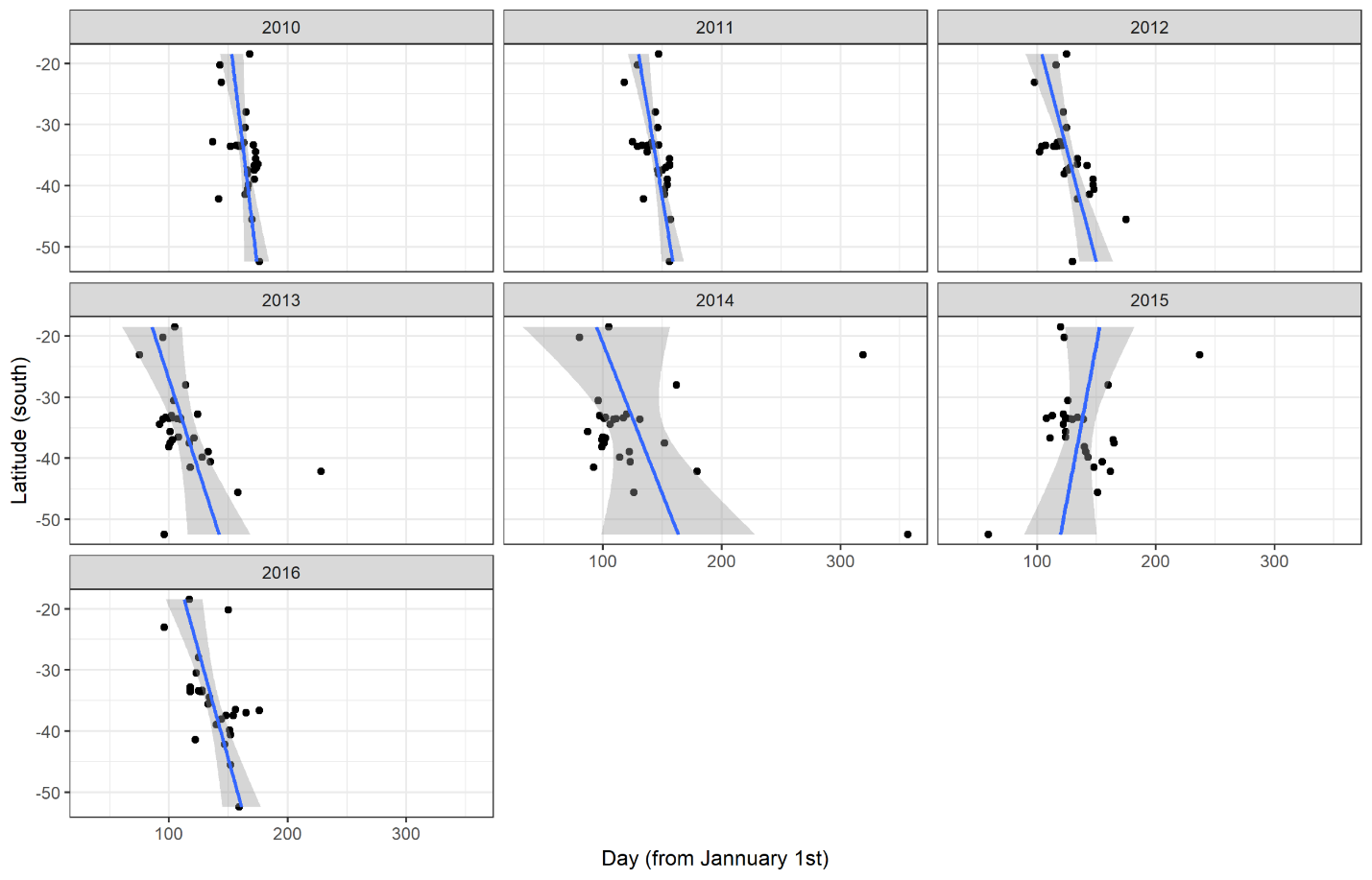


Linear model for start day vs latitude. Black dots represent Health Networks, blue lines the linear relation between latitude and start day and grey zones denote 95% CI.

**References**

1. Torrence C, Compo GP. A practical guide to wavelet analysis. Bulletin of the American Meteorological society **1998**; 79:61-78.

2. Van Panhuis WG, Choisy M, Xiong X, et al. Region-wide synchrony and traveling waves of dengue across eight countries in Southeast Asia. Proceedings of the National Academy of Sciences **2015**; 112:13069-74.

3. Choisy M, Rohani P. Changing spatial epidemiology of pertussis in continental USA. Proceedings of the Royal Society of London B: Biological Sciences **2012**; 279:4574-81.

4. Cazelles B, Chavez M, De Magny GC, Guégan J-F, Hales S. Time-dependent spectral analysis of epidemiological time-series with wavelets. Journal of the Royal Society Interface **2007**; 4:625-36.

5. Cazelles B, Chavez M, Berteaux D, et al. Wavelet analysis of ecological time series. Oecologia **2008**; 156:287-304.

6. Grinsted A, Moore JC, Jevrejeva S. Application of the cross wavelet transform and wavelet coherence to geophysical time series. Nonlinear processes in geophysics **2004**; 11:561-6.
